# Exploitation of Apoptotic Regulation in Cancer

**DOI:** 10.3389/fimmu.2018.00241

**Published:** 2018-02-27

**Authors:** David S. Ucker, Jerrold S. Levine

**Affiliations:** ^1^Department of Microbiology and Immunology, University of Illinois College of Medicine, Chicago, IL, United States; ^2^Department of Medicine, Division of Nephrology, University of Illinois College of Medicine, Jesse Brown Veterans Affairs Medical Center, Chicago, IL, United States

**Keywords:** tumorigenesis, apoptosis, microenvironment, selective adaptation, inflammation, immunity

## Abstract

Within an organism, environmental stresses can trigger cell death, particularly apoptotic cell death. Apoptotic cells, themselves, are potent regulators of their cellular environment, involved primarily in effecting homeostatic control. Tumors, especially, exist in a dynamic balance of cell proliferation and cell death. This special feature of the tumorous microenvironment—namely, the prominence and persistence of cell death—necessarily entails a magnification of the extrinsic, postmortem effects of dead cells. In both normal and malignant tissues, apoptotic regulation is exerted through immune as well as non-immune mechanisms. Apoptotic cells suppress the repertoire of immune reactivities, both by attenuating innate (especially inflammatory) responses and by abrogating adaptive responses. In addition, apoptotic cells modulate multiple vital cell activities, including survival, proliferation (cell number), and growth (cell size). While the microenvironment of the tumor may contribute to apoptosis, the postmortem effects of apoptotic cells feature prominently in the reciprocal acclimatization between the tumor and its environment. In much the same way that pathogens evade the host’s defenses through exploitation of key aspects of innate and adaptive immunity, cancer cells subvert several normal homeostatic processes, in particular wound healing and organ regeneration, to transform and overtake their environment. In understanding this subversion, it is crucial to view a tumor not simply as a clone of malignant cells, but rather as a complex and highly organized structure in which there exists a multidirectional flow of information between the cancer cells themselves and the multiple other cell types and extracellular matrix components of which the tumor is comprised. Apoptotic cells, therefore, have the unfortunate consequence of facilitating tumorigenesis and tumor survival.

## Introduction

A predisposition to apoptotic death among cells that have acquired a malignant mutation serves to facilitate one of the body’s primary defenses against cancer. Induction of apoptosis is a failsafe and occurs as a result of the tight interweaving of the multiple genes and signaling pathways regulating survival, proliferation, and growth (1–4). Were the cell’s demise the sole consequence of this defense against cancer, the benefits to the organism would be unambiguous and unopposed.

Apoptosis, while a cell-autonomous process, has postmortem consequences that are not simply cell-intrinsic. Through an array of mechanisms, both direct and indirect, dead or dying cells actively and potently influence other cells within their environment (5–8). Although earlier studies focused on the ability of apoptotic cells to suppress inflammation (9–12), apoptotic cells also affect a broad range of cellular functions, including such vital activities as survival (13–15), proliferation (13–15), differentiation (16), metabolism (17), and migration (5, 7, 8). Moreover, these effects are not only limited to the professional phagocytes charged with the clearance of apoptotic cells, but extend also to virtually every living cell in the vicinity of the apoptotic cell, regardless of its origin or lineage (14–20).

**Figure 1:**
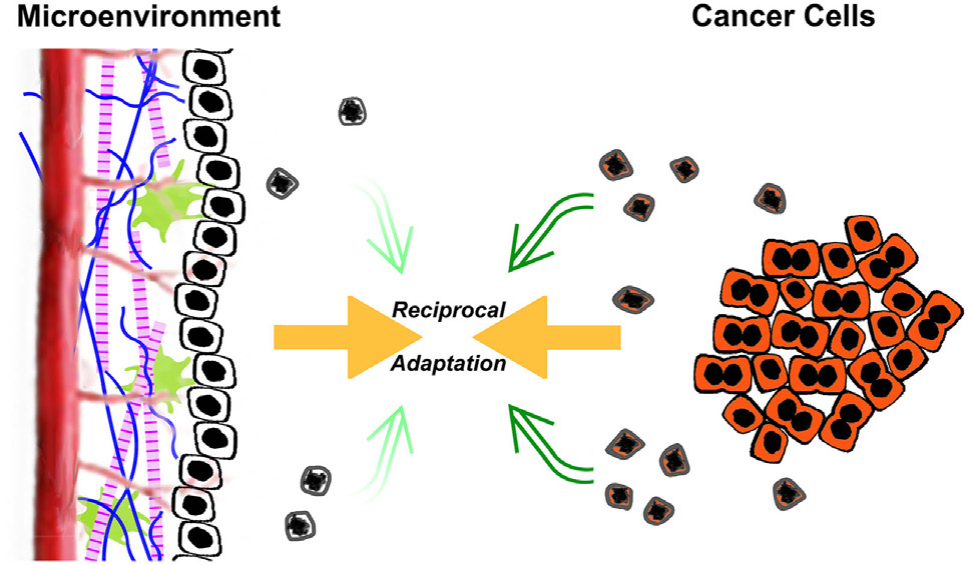
The reciprocal acclimatization of cancer cells and their microenvironment is enhanced by apoptotic cells. A dynamic flow of information exists between transformed cancer cells and the tumorous microenvironment in which they reside. The microenvironment consists of non-transformed stromal cells, both resident and recruited, phagocytic cells including macrophages, as well as extracellular matrix components. As depicted in the figure, the ongoing interaction between transformed cancer cells (shaded in orange) and their microenvironment leads to a process of reciprocal adaptation, in which the environment becomes progressively more conducive to cancer cell growth, and the cancer cells themselves become progressively more adapted to their environment. Through their postmortem effects, apoptotic cells (schematized here as shrunken and misshapen, with extensive nuclear condensation) impact tumorigenesis and tumor growth. The relative effects of transformed cells and their microenvironment on tumorigenesis likely are in a continuously dynamic balance. Certainly, the balance of those inputs changes during the life of a tumor. One obvious shift occurs following antitumor therapy (e.g., chemotherapy and radiotherapy), in which the rates of cell death increase dramatically, including both transformed cancer cells and non-transformed stromal cells. These elevated levels of apoptosis can lead to further tumor-promoting enhancement of the microenvironment.

It is owing to these extrinsic, postmortem effects of apoptosis that dying cancer cells may act to promote, rather than retard, tumorigenesis. This facilitation comes about through a subversion of normal homeostatic mechanisms. In much the same way that pathogens evade the host’s defenses through clever disguise and manipulation of key aspects of innate and adaptive immunity, cancer cells subvert several normal homeostatic processes, in particular wound healing and organ regeneration, to transform and overtake their environment (4, 16, 21–23). In understanding this subversion, it is crucial to view a tumor not simply as a clone of malignant cells, but rather as a complex and highly organized structure in which there exists a multidirectional flow of information between the cancer cells themselves and the multiple other cell types and extracellular matrix components of which the tumor is comprised (24). In a sense, a reciprocal process of acclimatization takes place, in which the environment becomes progressively more conducive to cancer cell growth, and the cancer cells themselves become progressively more adapted to their environment (Figure 1).

Several features unique to the milieu of the cancerous micro-environment facilitate this reciprocal adaptation, not least of which is the very prominence and persistence of cell death in nearly all cancers (4, 24, 25). As compared with normal tissues, tumors are characterized by increased rates of both proliferation and death, with tumor mass increasing if the rate of proliferation exceeds that of death (1–4). Increased cell death is not solely due to the failsafe induction of apoptosis during unregulated proliferation (1–4). Multiple other factors contribute to the death of cancer cells, including antitumor immune responses, competitive interactions among different clones, and metabolic stress as a result of limitations in growth factors, nutrients, and oxygen. While a variety of types of cell death arise, apoptosis is the predominant form of cell death in tumors, as it is in normal homeostatic physiology. Importantly, the rapidity and efficiency with which dead cells are cleared means that, in the absence of specific histologic labeling, cell death often goes undetected (4, 5, 7, 8). Even the heightened numbers of dead cells typically observed in cancerous tissues represent an underestimate of the actual number of dying cells. Indeed, with the possible exceptions of embryogenesis or the immature thymus, the degree of cell death in tumors far outstrips that found in any organ under physiological conditions. Moreover, the supply of dead cells undergoes more or less continuous renewal. This is unlike non-cancerous tissues, for which, even following severe injury, cell death rarely continues unabated for weeks to months, as occurs characteristically within growing tumors. This special feature of the tumorous microenvironment—namely, the prominence and persistence of cell death—necessarily entails a magnification of the extrinsic, postmortem effects of dead cells. In turn, this enables tumors to usurp and manipulate for their own advantage a number of the normal homeostatic processes initiated by the recognition and clearance of dead cells. As a result, cell death has the potential to be among the most crucial factors impacting the development and progression of cancers.

In this review, we will describe how, within the context of tumorigenesis, several of the normal homeostatic responses triggered by apoptotic cells are subverted, with the unfortunate consequence of facilitating tumorigenesis. At least in part, the reciprocal adaptation between cancer cells and their environment may be said to occur under the specific aegis of apoptotic cells. Because this seemingly paradoxical outcome of cell death derives mainly from the postmortem effects of apoptosis, we will focus on this aspect of cell death, rather than on the myriad genes involved in the regulation and execution of the apoptotic death program, many of whose mutations predispose to malignancy. Finally, we will highlight what, from our perspective, are some of the field’s major unanswered questions and areas open for investigation.

## Pathogenic Exploitation of Apoptotic Immunity

Infections with microbial pathogens provide a useful context in which to appreciate the subversion of postmortem apoptotic effects. In the acute context of pathogenic infection, the repertoire of potent immunosuppressive responses elicited normally by apoptotic cells (“Innate Apoptotic Immunity”; refs. 9–12, 18, 19) appears to be specifically exploited as a means of enhancing pathogenicity (26). Multiple microbial pathogens subvert the processes of apoptosis and Innate Apoptotic Immunity in this way.

One of the hallmarks of this pathogenic sabotage is that pathogens trigger the apoptotic cell death of cells that are expendable for productive infection. In other words, while those viable cells are not essential to the pathogen, the apoptotic corpses of those cells serve to enhance pathogenicity. For example, in the case of the lethal food-borne bacterial pathogen *Listeria monocytogenes*, the extensive induction of apoptotic cell death, especially among lymphocytes (27), is of particular interest. Lymphocytes are not critical for the *in vivo* replication of the bacterium, do not serve as substantial bacterial reservoirs, and are not the primary cells of entry for productive infection (28, 29). Still immunocompromised mice genetically deficient in lymphocytes are less susceptible to *L. monocytogenes* infection than are lymphocyte-replete, wild-type mice (30). The reconstitution of normal lymphocyte populations in these mutants restores pathogen susceptibility to wild-type levels (30). Strikingly, exogenous apoptotic lymphocytes, including uninfected apoptotic lymphocytes, are as effective as viable lymphocytes (29). Thus, although viable lymphocytes are dispensable for *L. monocytogenes* replication, apoptotic lymphocytes are important for *L. monocytogenes* pathogenesis (29). Because apoptotic cells are not susceptible to *L. monocytogenes* infection (29), the uptake of those apoptotic cells cannot be responsible for pathogen spread. Similar results have been obtained with a sepsis model of bacterial pathogenicity (31, 32). The specific action of apoptotic lymphocytes in these cases appears to be the suppression of host inflammation *via* Innate Apoptotic Immunity.

Another hallmark of this process is that pathogen-induced host cell apoptosis is dissociable from the postmortem effects of the apoptotic cells. Again, in the case of *L. monocytogenes*, lymphocyte apoptosis depends upon pathogen-dependent stimulation of host innate immunity (and production of interferon-β; refs. 33–35), as well as a pathogen-encoded pore-forming protein (Listeriolysin *O*; ref. 36). The efficacy of apoptotic cells is fully independent of these mediators, however.

In the chronic setting of a tumor, the consequences of cell death (as judged by interference with that process; see below) allow the suggestion that apoptotic cells do similar things. Just as microbial pathogens exploit Innate Apoptotic Immunity, the extrinsic, postmortem effects of apoptotic cells also appear to be exploited in tumorigenesis. Indeed, it may even be that in the more chronic tumorigenic setting, a broader spectrum of postmortem apoptotic effects is involved.

## Clinical Evidence of the Countervailing Role of Apoptosis in Cancer

Initial evidence for a paradoxical, tumor-enhancing role of apoptosis in cancer arose from multiple studies of clinically related cohorts across a broad spectrum of cancers in which an association was observed between the extent of apoptosis and the aggressiveness of the underlying malignancy (22, 37–57). Cancers studied included non-Hodgkin’s lymphoma (37, 38), synovial sarcoma (39), and carcinomas of the tongue (40), esophagus (41), bladder (42, 43), breast (22, 44–46), endometrium (47), prostate (48, 49), cervix (50–52), kidney (53), stomach (52, 54), liver (55), ovaries (52, 56), larynx (57), colorectum (52), and head and neck (22). In these studies, a statistical correlation was observed between the extent of apoptosis and the following parameters: histologic grade (37–39, 42–45, 53–55), cancer stage (39, 43, 55), mitotic/proliferative index (37, 38, 43–45, 50, 55, 56), metastasis (40, 45, 55), mortality (22, 37, 38, 43–46, 50, 52, 56, 57), recurrence following treatment (22, 40, 43–46, 49–51), local invasiveness (41, 55), tumor progression (43, 48), and tumor size (39, 45). Moreover, while a statistical association does not necessarily imply causality, it is noteworthy that in several of these studies, upon multivariate analysis, an index of the extent of apoptosis proved to be one of very few independent predictors (or even the only independent predictor) of overall or disease-free survival (37, 39, 43, 49–51, 55, 57).

## The Language of Apoptotic Cells

The increased rate of cell death typical of nearly all cancers means that the specialized microenvironment of tumors, more so than other tissues under physiologic conditions, is characterized by a robust flow of information centered on dead or dying cells—*into* malignant cells from their environment that influences their decision whether to live or die, and *out of* them to live cells in their vicinity, both cancerous and non-cancerous (Figure 1). A sense of the vast extent of apoptosis observed in human malignancies can be informative. In most studies of human cancer, apoptosis has been quantified in the form of an apoptotic index, defined as the number of apoptotic nuclei per 100 intact neoplastic cells (37–42, 46, 47, 49–56). While rigor varied widely across these studies, the mean apoptotic indices in general fell in the range of 0.5–2.0% (37–39, 41, 42, 46, 49, 50, 56). With increasing markers of tumor aggressiveness, apoptotic indices reached as high as 5–10% (40, 49, 51, 54, 55), and at times even exceeded 10% (53). These numbers offer powerful evidence of the markedly increased rates of apoptosis characteristic of most tumors. While apoptotic cell death may be largely invisible under physiologic conditions (4, 5, 7, 8), it is not silent.

Transmission of information from apoptotic cells to the environment occurs in one of two fundamental ways, either directly, through physical interaction between dead and live cells, or indirectly, without physical interaction. Direct effects occur most commonly *via* receptor-mediated recognition by live cells of adjacent dead cells or their fragments (5–8, 11, 14, 18, 19). Indirect effects are most frequently the result of soluble mediators released from the dying cells, but can entail more subtle mechanisms (4–8). For example, apoptotic cells may adsorb soluble mediators and thereby lower effective concentrations, precluding viable cell responses (58). Dying cells also may shed various membrane-enclosed vesicles containing a combination of cytosolic proteins, RNA, and lipids (59–61) that can serve in information transmission. Depending upon the origin of these extracellular vesicles, whether from the plasma membrane or endosomes, they are referred to as microparticles or exosomes, respectively (62). Docking of these vesicles at the surface of live cells, followed by their fusion with the plasma membrane, or by their endocytosis and fusion within an endocytic compartment, leads to release of their contents and delivery of the message those contents represent (62).

A striking range and complexity characterize all steps of the information flow from apoptotic cells to live cells in their vicinity (4–8). Viewed as a language, dead cells carry a surprisingly large amount of information, with an extensive vocabulary and an intricate grammar. While this complexity has been shown predominantly for non-tumorous cells and tissues, some data suggest the same is true for tumors, especially since the ability to recognize and respond to dead cells seems to be ubiquitous across practically all organs and cell lineages (18, 63, 64). Death-related variables from which information can be extracted include, but are by no means limited to, the mode of cell death (11, 14, 15, 17, 65)—and, under certain circumstances, perhaps even the conditions and the particular inducer of that form of cell death (15, 66)—as well as the pattern, distribution, kinetics, rate, and extent of cell death (67–69). Moreover, the response by any given live cell, whether cancerous or not, depends as much on the identity of the responding cell itself—its lineage (13–15), organ of residence (13–15), and stage of differentiation (70, 71)—as on the specific nature of those death-related variables. It is easy to imagine that even live cells lacking direct physical interaction with apoptotic cells or their released mediators may be affected by the dynamic multidirectional flow of information. A ripple effect may ensue, in which apoptotic cells stimulate the synthesis and release of cytokines and mediators from live cells, and in turn these cytokines and mediators then modulate the activity of other live cells that reside at a distance from the dead cells, at the fringe or beyond the tumorous microenvironment, even in distal organs and tissues.

## Paradigm

As first formulated in an influential review on cancer (2, 3), the evolution of a cell from normal to neoplastic entails the acquisition of as many as eight discrete biological capabilities. These capabilities, or hallmarks, represent a series of steps, each of which confers a trait or selective advantage necessary to the emergence of a clone of highly malignant cells. Hallmarks include: (i) sustained proliferative signaling, (ii) evasion of growth suppressors, (iii) resistance to cell death, (iv) induction of angiogenesis, (v) tissue invasion and metastasis, (vi) reprogramming of energy metabolism, (vii) evasion of immune destruction, and (viii) replicative immortality. While the succession of hallmarks need not occur in any set order, the overall process itself proceeds in a step-by-step manner analogous to that of other cases of natural selection.

Integral to this evolution is the interaction between cancer cells themselves and the microenvironment in which they reside. The tumorous environment is a specialized structure, consisting not only of malignant clones, but also of multiple non-transformed cell types, both resident and recruited (2–4, 24, 25). These ostensibly normal cells, together with a surrounding extracellular matrix, comprise the tumor-associated stroma. A critical determinant underlying the acquisition of hallmark capabilities is the genomic instability of cancer cells (2, 3). The facilitated occurrence of genetic mutations and/or epigenetic changes affecting gene expression catalyzes the development of heritable phenotypes better adapted to the tumorous environment (2, 3, 72). In turn, cancer cells contribute to the favorable transformation of their environment (4, 24, 25). Through tissue remodeling and modulation of the function of stromal cells, such as macrophages (4, 73–75), cancer cells help make the environment more conducive to their outgrowth and supremacy.

Increasingly, apoptotic cells have been recognized as participants in the reciprocal adaptation between cancer cells and their microenvironment (4). While an established role for apoptotic cells has so far been limited to only a few of the hallmarks—most extensively, in sustained proliferative signaling and evasion of growth suppressors (22, 74–80)—it is striking how many of the biological capabilities of cancer cells are known to be impacted by apoptotic cells under non-cancerous conditions. Indeed, the only one of the eight hallmarks for which a convincing, or at least highly suggestive, example of the influence of apoptotic cells does not yet exist is the induction of replicative immortality.

Most of these activities of apoptotic cells fall under the rubric of two homeostatic processes in which the active role of apoptotic cells has been carefully explored, namely, wound healing and organ regeneration (4, 21, 67–69). Although these two processes probably differ more quantitatively than qualitatively, they do represent clearly separable stages in the life of a tumor, with treatment representing the boundary. As first pointed out over 30 years ago (21), and expanded upon by numerous reviewers since (4, 81, 82), growing tumors are like “wounds that do not heal,” or even “wounds that do not stop repairing,” whereas the repopulation of a tumor that occurs following therapy most resembles organ regeneration. These broad similarities dwarf the finer differences and provide a convenient lens through which to view the subversion of homeostatic processes by the apoptotic cells within tumors.

## Apoptotic Cells and the Hallmarks of Cancer

**Figure 2:**
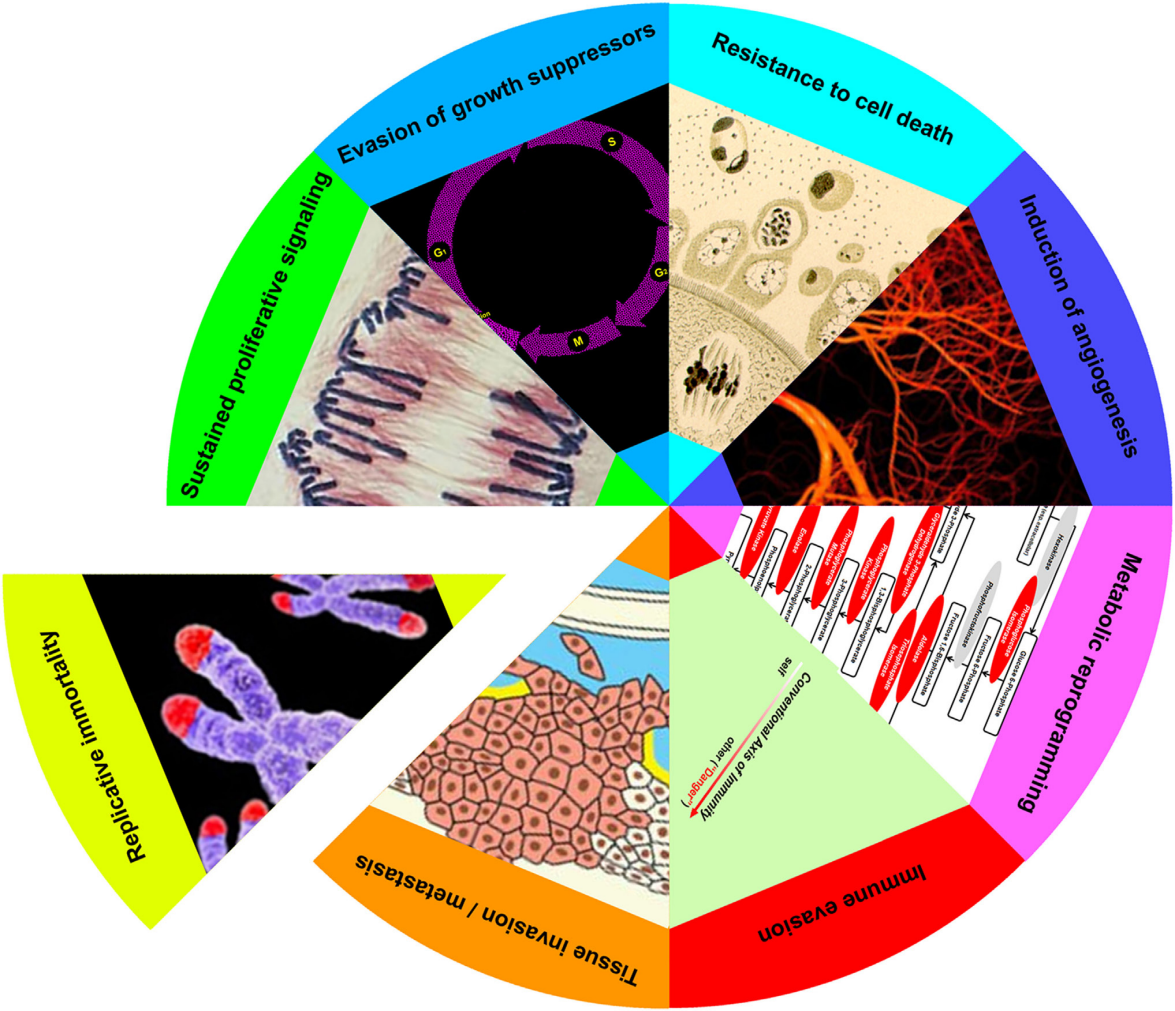
Apoptotic cells enhance achievement of cancer hallmarks. Eight hallmarks of cancer (sustained proliferative signaling, evasion of growth suppressors, resistance to cell death, induction of angiogenesis, metabolic reprogramming, immune evasion, tissue invasion/metastasis, and replicative immortality) have been elaborated (3). With the exception of the last, apoptotic cells enhance each of these cancer attributes, as discussed in the text.

Postmortem apoptotic modulation targets almost every hallmark of cancer (Figure 2). The sole hallmark for which no evidence of apoptotic modulation yet exists is replicative immortality, in which cancer cells escape their inbuilt limitation on replicative doublings. This may not be so surprising since, given the increased numbers of apoptotic cells observed in nearly all cancers (4, 24, 25), a heightened effect of apoptotic signaling in cancerous as opposed to non-cancerous tissues would be expected. Moreover, as many of the hallmarks of cancer represent unregulated and essentially continuous manifestations of cellular functions normally modulated by apoptotic cells, it is tempting to hypothesize a prominent role for apoptotic cells in hallmark acquisition. As examples of the ways in which tumors can subvert the normal homeostatic functions of apoptotic cells, we examine the many parallels between tumor progression and the processes of wound healing and organ regeneration.

### Tumor Growth and Progression: Wounds That Will Not Heal

**Figure 3:**
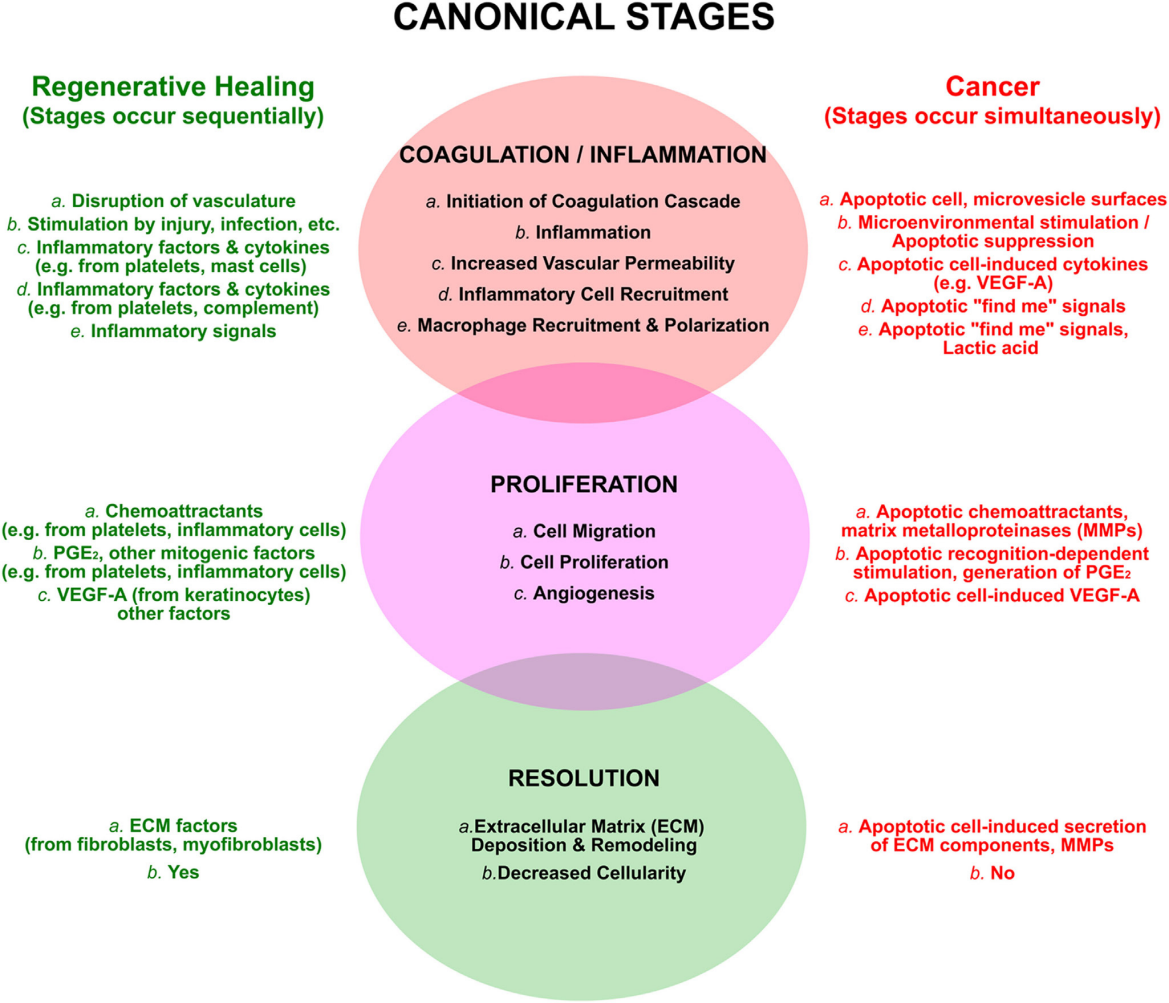
The processes of regenerative wound healing are subverted by apoptotic cells in cancer. The canonical attributes of wound healing are categorized into overlapping and sequential phases, as discussed in the text. These are compared to similar, yet distinct, events occurring in an ongoing and simultaneous manner in cancer, resulting largely from the postmortem effects of apoptotic cells. The view that cancers are “wounds that do not resolve” emerges from this perspective.

The tissue repair that follows a wound can be divided into three broad and overlapping phases (Figure 3; see refs. 21, 81, 82). In the first inflammatory phase, a blood clot is formed and seals the wound; local inflammation, a direct consequence of injury, leads to the recruitment of inflammatory cells. In the second phase, new tissue, called granulation tissue, is formed and replaces the blood clot. Macrophages are key players in this phase, which entails the formation of new blood vessels (an especially critical event in the case of tumors), the laying down of new extracellular matrix, and an overall increased proliferation of multiple cell types, including fibroblasts and keratinocytes. During the final phase, granulation tissue is converted into a scar, with an overall decrease of cellularity and an extensive remodeling of the extracellular matrix. For each of these phases of wound healing, there are parallels with tumor growth, and for each of these parallels a plausible role for apoptotic cells exists.

The blood clot, formed during the initial inflammatory phase of wound healing, serves multiple purposes. It provides a protective barrier against infection and evaporative loss, it serves as a reservoir of growth factors, and it acts as a scaffold for the multiple cell types attracted to the wound. The two major matrix components of a clot are fibrin and fibronectin. Fibrin is generated as an end product of the coagulation cascade, whereas fibronectin leaks through capillary walls because of a local increase in vascular permeability. Although the origins of fibrin and fibronectin differ in the case of cancer, most tumors are also characterized by the presence of a fibrin and fibronectin matrix (21, 81). Apoptotic cells influence the generation and localization of both of these proteins. By virtue of reorganization of their membrane phospholipids (83, 84), apoptotic cells, and especially their shed microvesicles (85), are procoagulant, thereby providing a nidus for initiation and amplification of the coagulation cascade. In addition, exposure to apoptotic cells upregulates the expression by macrophages and endothelial cells of vascular endothelial growth factor (VEGF)-A, a factor that potently augments vascular permeability (73, 86, 87).

A key event in the initiation of coagulation is a loss of membrane asymmetry in platelets and other blood cells (88). Exoplasmic exposure of several phospholipids normally retained within the cytoplasmic leaflet of cell membranes, such as the anionic phospholipid phosphatidylserine (PS) and the amphipathic phospholipid phosphatidylethanolamine, supports the binding of clotting factors and initiates the coagulation cascade (88, 89). A similar loss of membrane asymmetry occurs in cells undergoing apoptosis (90, 91). Exposure of PS is mediated, generally, by activation of a phospholipid scramblase (an energy-independent bidirectional transporter that dissipates membrane asymmetry) and/or inhibition of a phospholipid flippase (a P4-ATPase that enhances asymmetry by transporting specific membrane phospholipids against their concentration gradient; refs. 89–91). The procoagulant activity of apoptotic cells, and especially their shed microvesicles (85), may, therefore, be attributed largely to their mimicking of the surface membranes of activated platelets and other blood cells. Although the normal physiologically handling of apoptotic cells does not appear to predispose to clotting disorders, the prominence and persistence of apoptotic death within tumors may upset the delicate balance between pro- and anti-coagulant activities.

Closely related to its procoagulant function, the exposure of PS on the outer leaflet of the apoptotic cell membrane has been identified as one element involved in the recognition of apoptotic cells. A related group of receptors with intrinsic tyrosine kinase activity, composed of the molecules *T*yro3, *A*xl, and *M*ertk (the “TAM receptor” family) are involved in PS-dependent apoptotic recognition (92, 93). TAM receptor-mediated recognition of apoptotic cells relies upon Vitamin K-dependent bridging molecules and is associated with the phagocytic clearance of apoptotic cells. TAM receptors are expressed primarily on myeloid cells of the immune system and exert a variety of immunoregulatory and other functions, including the suppression of inflammation and the enhancement of angiogenesis (92, 94, 95).

The recognition of apoptotic cells also occurs by PS-independent mechanisms (18, 96), relying on surface-exposed protein determinants (97). This recognition, which is not encumbered by serum-derived tethering molecules (18), triggers immediate-early responses independent of phagocytosis (11, 14, 15, 17). These include all of the homeostatic processes subverted in cancer (Figure 2). Most notably, PS-independent apoptotic recognition is ubiquitous among all cell types (18).

### Tumor-Associated Macrophages (TaMacs)

Among the inflammatory cells recruited to wounds during the initial inflammatory phase are neutrophils, mast cells, and macrophages (21, 81, 82). Of these, the macrophages are the one whose pivotal role in wound healing and the acquisition of many of the hallmarks of cancer is best established (4, 73–75, 98–100). TaMacs acquire a phenotype that favors proliferation, angiogenesis, tissue invasion and metastasis, and evasion of immune destruction (4, 73–75, 98–100). Like macrophages in healing wounds, TaMacs are intimately involved in remodeling of the extracellular matrix and in creating an environment more favorable to tumor growth (4, 74, 75). Indeed, of the many parallels between wound healing and cancer, one of the strongest is the molecular concordance in gene expression patterns between TaMacs and repairing tissues (74, 75, 81).

Far more controversial is the origin and state of polarization of TaMacs, as compared with macrophages in other tissues and other pathological conditions. There is a general consensus that TaMacs are recruited from the blood as monocytic precursors (98–100). In the case of a murine mammary tumor model, TaMacs were shown to be derived predominantly from C–C chemokine receptor type 2 positive (CCR2^+^) monocyte precursors, and were functionally and phenotypically distinct from the tissue macrophages of non-cancerous glands (101). As shown in two other models, murine breast cancer and xenografted Burkitt’s lymphoma, TaMacs had a higher proliferative capacity than resident macrophages and, therefore, required less replenishing from the blood (74, 101). Although from a functional perspective, TaMacs found in most tumors resemble alternatively activated (M2-like) more than classically activated (M1 or M_(IFN- γ/LPS)_) macrophages (98–100), their gene expression patterns, when carefully examined, fit the pattern of neither macrophage subtype (74, 101). For example, unlike alternatively activated macrophages, TaMacs from mouse mammary tumors lacked IL-4 dependence and failed to express several characteristic M2-like genes (101). Correspondingly, TaMacs from Burkitt’s lymphoma expressed multiple classically activated genes (74). While macrophage activation is best viewed within a continuum of gene expression patterns rather than fixed in discrete polarized states, these data nonetheless suggest the presence within the tumor microenvironment of unique determinants influencing the phenotypic and genotypic state of TaMacs. One of these determinants, as demonstrated in murine models of melanoma and lung carcinoma, is lactic acid, which is produced by cancer cells as a result of their relatively hypoxic microenvironment (73). The effect of lactic acid is mediated by the transcription factor hypoxia-inducible factor 1α (HIF-1α), which induces strong expression of the genes for VEGF-A and arginase 1 (73). Again, non-tumor wounds also are relatively hypoxic environments, so that at least some of the similarities in gene expression patterns between TaMacs and wound tissue may be attributable to the effects of lactic acid and HIF-1α (21, 81, 82).

Significantly, exposure to apoptotic cells contributes to the phenotypic and genotypic expression of a number of the pro-oncogenic properties of TaMacs. *In vitro* exposure of classically activated M_(IFN- γ/LPS)_ macrophages to apoptotic lymphoma cells shifted their gene expression pattern toward that of *in situ* TaMacs, as obtained by laser-capture microdissection of Burkitt’s lymphoma xenografts (74, 75). Among the gene clusters identified in TaMacs *in situ* were several specifically associated with tumor progression and wound healing. These included functional clusters related to the key cancer hallmarks of proliferation, cell death, and differentiation; extracellular matrix deposition and remodeling; and angiogenesis (74, 75, 81, 82). In this same model, suppression of apoptosis, *via* expression of the anti-apoptotic genes Bcl-2 or Bcl-X_L_, led to reduced TaMac accumulation and reduced angiogenesis when lymphoma cells were xenografted into SCID mice (74). Somewhat surprisingly, suppression of apoptosis had a minimal effect on tumor growth *in vivo*, despite promoting expansion *in vitro* (74). This may be related to the reduced angiogenesis observed in apoptosis-suppressed xenografts or to differences between *in vitro* and *in vivo* growth conditions. Similarly suggestive, though less complete, evidence for a role of apoptotic cell-dependent effects on TaMacs also was found in a model of melanoma (74).

Supportive data for the importance of apoptotic cells in the conditioning of TaMacs also comes from models of prostate and breast cancers (102, 103). Coculture of bone marrow derived macrophages with apoptotic cells from several prostate cancer cell lines induced an M2-like state of macrophage polarization characterized by increased expression of multiple M2-like-associated genes without alteration in the expression of several markers of classical activation (102). This occurred in a milk fat globule-EGF factor 8 (MFG-E8)-dependent manner, with significantly increased levels of MFG-E8 detected in exosomes from prostate cancer cells (102). Additional provocative findings were reported in a model of breast cancer, in which the widespread apoptosis of mammary epithelial cells that occurs in the postpartum period was shown to enhance tumor metastasis (103). Molecular or pharmacologic blockade of the clearance of apoptotic cells led to a reduction of M2-like TaMacs (without a change in the total number of macrophages) and a concomitant reduction in tumor metastasis as compared to nulliparous mice (103).

### Tumors As Continually Regenerating Organs

The direct enhancement of cancer cell proliferation and tumor growth by apoptotic cells may be the most well-established postmortem apoptotic effect. More than 50 years ago, it was first reported that admixing lethally irradiated cancer cells with live cancer cells led to a higher incidence of rapidly growing tumors and shorter survival times when compared to injection of an equal number of live cancer cells (79). These results have been replicated both *in vitro* and *in vivo* in a variety of cancers and cancer cell lines (22, 74–80). In one study, the effect was shown to be specific to apoptotic cells, as necrotic cells had no effect (76). A strong parallel exists with wound healing in that the proliferative effect of apoptotic cells in both cancer and wound healing strongly depended on caspase-3-mediated activation of calcium-independent phospholipase A2 (iPLA_2_) and the iPLA_2_-mediated generation of prostaglandin E_2_ (PGE_2_; refs. 16, 22, 52, 77, 78). An important consideration here is that, in these experimental studies, the number of apoptotic cells typically was very large in comparison to that of viable cells, with the ratio of apoptotic to viable cells varying from 20–50:1 to as much as 1,000–10,000:1 (22, 76–80). In one study, the precise ratio proved important, as the effect was found to be highly dependent on apoptotic dose (80).

The later phases of wound healing involve extensive remodeling of the extracellular matrix (21, 81, 82). While the deposition and renovation of connective tissue is time-limited in wounds, eventually leading to the formation of a healed scar, the process is ongoing in tumors. Nevertheless, the genetic signatures of wounds and tumors—and, in particular, of TaMacs—are very similar, highlighting their many shared features (73–75, 81, 82). Among the categories of mutually expressed genes are proteinases and their regulators involved in the cleavage and restructuring of extracellular matrix components [metalloproteinase (MMP)-2, MMP-3, MMP-12, tissue inhibitor of MMP-2 (TIMP2), urokinase plasminogen activator], components of the extracellular matrix (fibronectin-1), and various cytokines regulating the activity and state of differentiation of fibroblasts, myofibroblasts, and other stromal cells (platelet derived growth factor, transforming growth factor-β, VEGF; refs. 73–75, 81, 82).

Antitumor therapies, of course, trigger massive cell death responses. While clearly an intended and vital outcome of therapy, the abrupt upsurge of cell death following radiation or chemotherapy is a double-edged sword, holding the potential to undermine the direct benefit of therapy by promoting the proliferation of surviving tumor cells (22, 23, 77, 78). The repopulation of tumors following therapy may be compared to the compensatory proliferation and organ regeneration observed in lower organisms following structural injury or amputation (67–69). In humans, the only organ with similar regenerative capacity is the liver (16). Many of the factors and signaling events involved in tumor repopulation mirror those driving organ regeneration in lower organisms as well as those promoting the pre-treatment growth and progression of tumors in mice and humans (22, 67–69, 74–80, 104–106). For example, caspase-3-mediated cleavage of iPLA_2_, and the subsequent generation of PGE_2_, which has been shown to be important for the proliferative effect of apoptotic cells in wound healing and tumor growth, also play a critical role in liver regeneration (16) and tumor repopulation (22, 23, 77, 78). The potential importance of this pathway in human cancer is highlighted by an association between elevated levels of caspase-3 and several markers of tumor aggressiveness, including shortened survival, in a variety of cancers (22, 52).

In many ways, the role of apoptotic cells in tumor regeneration may be viewed as an exaggeration of their role in pre-treatment tumor growth and progression. Still, there may be several notable differences. For example, the massive increase of cell death after therapy shifts the proportions of live and dead cells, so that apoptotic cells almost certainly pass from a minority to a majority of cells. The extremely elevated ratios of apoptotic to viable cells used in most studies describing a proliferative effect of apoptotic cancer cells actually may reflect the post-therapy situation (22, 76–80). Additionally, since therapy-induced death is not limited to cancerous cells, the bystander death of non-cancerous cells within the tumor’s microenvironment may have further deleterious consequences. It may be that the activities of cancerous and non-cancerous apoptotic cells differ. For example, apoptotic human umbilical vein endothelial cells (HUVEC) stimulated the growth *in vitro* of glioma cell lines; as with apoptotic cancer cells, the effect was linked to PGE_2_ released from apoptotic HUVEC (78). Similar results were obtained *in vivo* in murine models of fibrosarcoma and melanoma (107). Following therapy, tumors not only grew twice as fast but also became resistant to further radiation in mice whose endothelial cells were rendered more sensitive to radiation-induced apoptosis (107). Further, non-cancerous cells need not die to have a profound impact on tumor repopulation (108, 109). Following chemotherapy, secreted factors produced by stressed stromal cells within the tumor’s microenvironment, which have sustained sublethal DNA damage, may enhance tumorigenesis and promote resistance to future therapy. For example, transcripts of the Wnt family member, wingless-type MMTV integration site family member 16 B (WNT16B), were increased approximately sixfold in prostate stroma after chemotherapy (109). Augmented expression of WNT16B within the prostate tumor microenvironment *in vivo* promoted cancer cell proliferation, migration, and tumor invasiveness, and attenuated the effects of chemotherapy (109). While this study did not specifically address the role of apoptotic cells, the induction of WNT16B by genotoxic stress and the subsequent role of Wnt-dependent signaling events in accelerated repopulation are reminiscent of the roles of apoptotic cells in organ regeneration and compensatory proliferation of lower multicellular organisms (67–69, 104–106).

### Immunity, Apoptosis, and Tumorigenesis

The impact of apoptotic cells in suppressing inflammatory responses (“Innate Apoptotic Immunity”) is well established (9–12, 18, 19). The contribution of inflammation appears to be critical in tumorigenesis, and the potential for apoptotic cell intervention on this level is obvious. A recent elegant series of studies sheds particular light on the role of inflammation in tumor initiation in the context of the liver (110–114). The combination of chronic inflammation and continuously increased rates of apoptotic hepatocellular death leads to the spontaneous development of hepatocellular carcinoma. While the liver may be unique in its susceptibility, perhaps because of its enormous regenerative capacity, abundant evidence supports a role for inflammation as an enabling feature of tumor development and as an essential characteristic of the tumor microenvironment (3, 4, 74, 75, 98, 100). On the surface, it may appear difficult to reconcile ongoing inflammation with the known potent anti-inflammatory properties of apoptotic cells (9–11), especially as they are present in tumors in increased numbers (4, 24, 25). In the case of wounds, a plausible resolution is that macrophages recruited during the initial inflammatory phase start out in a classically activated state [M1 or M_(IFN- γ/LPS)_] state, but, under the influence of apoptotic cells and other factors, they eventually transition to an alternatively activated M2-like state. Given that tumors “never heal,” it may be that pro- and anti-inflammatory forces are each continuously and potently present within tumors, and that their continuous opposition helps to explain the lack of a clear state of polarization among TaMacs (4, 73–75, 98–101).

While robust adaptive immunity certainly can function effectively against established tumors (as demonstrated dramatically with the recent success of T cell stimulatory treatments targeting so-called checkpoint inhibitors [such as PD-1 and its ligands]; ref. 115), the role of adaptive immunity normally in tumorigenic initiation and propagation is less well-defined. A plethora of studies spanning almost the entirety of the field of tumor immunology has led to the striking axiom that tumors, generally, are poorly immunogenic, eliciting feeble immune responses. The abundant presence of anti-inflammatory apoptotic cells within a tumor gives rise to the notion that apoptotic tumor cells may be responsible for triggering tumor-specific immunosuppression. However, in contrast to their well-described suppression of innate immune responses, the effects of apoptotic cells on adaptive immune responses are uncertain, even in the non-tumorigenic setting (116). Further, it is not clear whether a common basis exists for the poor immunogenicity of tumors. This might reflect a deficit in antigen presentation, a failure to activate antigen-specific T lymphocyte responders (for example due to a defect in co-stimulation), and/or the stimulation of antigen-specific inhibitory (e.g., T-regulatory) cells.

Under experimental conditions (especially *in vitro*), it is clear that apoptotic tumor cells can be a source of antigen and elicit antigen-specific T cell responses (116). Whether this is meaningful physiologically, and what the relative potency of apoptotic immunostimulatory activity might be, remains unresolved, although other work suggests that non-apoptotic corpses may be more immunogenic (117, 118). It is worth noting that, in cases in which apoptotic immunostimulatory activity has been observed, those apoptotic cells also have provided an innate immune stimulus (due to viral infection; refs. 119, 120). On the other hand, compelling data indicating that apoptotic cells interfere with the process of antigen presentation or skew T cell responses toward a regulatory phenotype are lacking, and the possibility that co-stimulatory molecule expression is modulated by apoptotic cells is contentious. Published studies have variously reported that the expression of one or more co-stimulatory molecules (CD40, C80, and CD86) is diminished, increased, or unaltered (118, 121–123).

Perhaps the most parsimonious view is that, within the tumor microenvironment, the preponderance of apoptotic cells, which shift TaMacs away from a classically activated profile and toward an alternatively activated one, has the potential to attenuate inflammatory responsiveness generally. In this context, requisite innate immune triggers for adaptive immune responsiveness may be insufficient. This perspective also suggests that the paradigmatic conviction that the tumor microenvironment is frankly pro-inflammatory may be in need of re-evaluation.

## Open Questions for Investigation

Our discussion of the multifaceted effects of apoptotic cells on tumors rests on the recognition of the unique and ongoing place of apoptotic cells in the tumor microenvironment. Implicit in our discussion is the assumption that the potent postmortem regulatory activities of apoptotic cells are comparable between transformed and non-transformed apoptotic cells. This, however, remains to be tested experimentally, not only in terms of the apoptotic cells eliciting a response, but also in terms of the responding cells themselves. In particular, the possibility exists that subtle differences distinguish the postmortem activities of transformed and non-transformed apoptotic cells. Similarly, it remains to be explored whether subtle differences also distinguish the repertoire of responses of viable transformed and non-transformed cells to apoptotic ones. We have noted that apoptotic cells modulate multiple vital cell activities of untransformed cells, including their survival, proliferation, and growth, but that no evidence exists for the apoptotic modulation of the cancer hallmark of replicative immortality. This is an interesting issue for exploration.

Just as apoptosis is ongoing throughout the life of a tumor, so too is inflammation. We have suggested that the preponderance of apoptotic cells in the tumor microenvironment may shift subtly its balance away from a pro-inflammatory one. While our understanding of the anti-inflammatory, homeostatic effect of apoptotic cells is best contextualized in terms of wound healing, there are important differences between healing wounds and tumors. For example, what are the long-term consequences of an “unhealing” tumor for apoptotic modulation? Might the resolving activity of apoptotic cells eventually become “exhausted” in this setting?

The basis of the insubstantial immunogenicity of tumors remains puzzling and is a critical issue for resolution. Certainly, the state of inflammation within the tumor microenvironment, and the question of whether apoptotic suppression of innate immune responsiveness is the basis of adaptive immune unresponsiveness, must be major considerations. A direct evaluation of the efficacy with which antigen-specific T cell responses (including cross-primed responses) are elicited by apoptotic tumor cells, as compared with other dead tumor cell forms, is urgently needed. In addition, it is important to know whether apoptotic modulation of adaptive immune responsiveness, like that of innate immune responsiveness, is manifest in a dominant manner.

While apoptosis is the primary mechanism by which cells die physiologically and, as we have discussed, is ongoing throughout tumor life, other forms of cell death can occur as well. Notably, and especially post-chemotherapy, this may include “immunogenic cell death” (6, 66). Cells dying in that way, in contrast to typical immunosuppressive apoptosis on which we have focused, can elicit immune responses. It will be interesting to consider the postmortem effects of those non-immunosuppressive cell death forms and the roles that they play in tumorigenesis.

## Author Contributions

DU and JL collaborated on the thesis of this review article, and wrote it together.

## Conflict of Interest Statement

The authors declare that the research was conducted in the absence of any commercial or financial relationships that could be construed as a potential conflict of interest.
